# Supplementary material for: Bora phosphorylation substitutes in trans for T-loop phosphorylation in Aurora A to promote mitotic entry
Source: Nat Commun. 2021 Mar 26;12:1899. doi: 10.1038/s41467-021-21922-w (PMC7997955; doi:10.1038/s41467-021-21922-w)
Supplement: Supplementary file 1 — Supplementary Information [file 41467_2021_21922_MOESM1_ESM.pdf]

Supplementary Information

Bora phosphorylation substitutes in trans for T-loop phosphorylation in Aurora A to promote mitotic entry

Tavernier N[1, #], Thomas Y[2], Vigneron S[3], Maisonneuve P[1], Orlicky S[1], Mader P[1], Regmi SG[4], Van Hove L[2], Levinson NM[5], Gasmi-Seabrook G[6], Joly N[2], Poteau M[7], Velez-Aguilera G[2], Gavet O[7], Castro A[3], Dasso M[4], Lorca T[3], Sicheri F[1, 8, 9] * and Pintard L[2, *]

[1]Centre for Systems Biology, Lunenfeld Tanenbaum Research Institute, Sinai Health System, Toronto, ON M5G 1X5, Canada

[2, #] Programme équipe Labellisée Ligue Contre le Cancer, Institut Jacques Monod, UMR7592, Université de Paris, CNRS, Paris, France

[3]Centre de Recherche de Biologie cellulaire de Montpellier, UMR 5237, Université de Montpellier, CNRS, 34293 Montpellier Cedex 5, France

[4]Eunice Kennedy Shriver National Institute of Child Health and Human Development, Bethesda, MD, United States

[5]Department of Pharmacology, University of Minnesota, Minneapolis, United States

[6]Princess Margaret Cancer Centre, University Health Network, Toronto, ON, M5G 1L7, Canada

[7]Institut Gustave Roussy CNRS UMR9019, Villejuif, France

[8]Department of Molecular Genetics, University of Toronto, Toronto, ON, Canada

[9]Department of Biochemistry, University of Toronto, Toronto, ON, Canada

* These authors contributed equally
# Present address

Correspondance:
sicheri@lunenfeld.ca
Lionel.pintard@ijm.fr

Supplementary Figures:

Tavernier et al. Figure S1

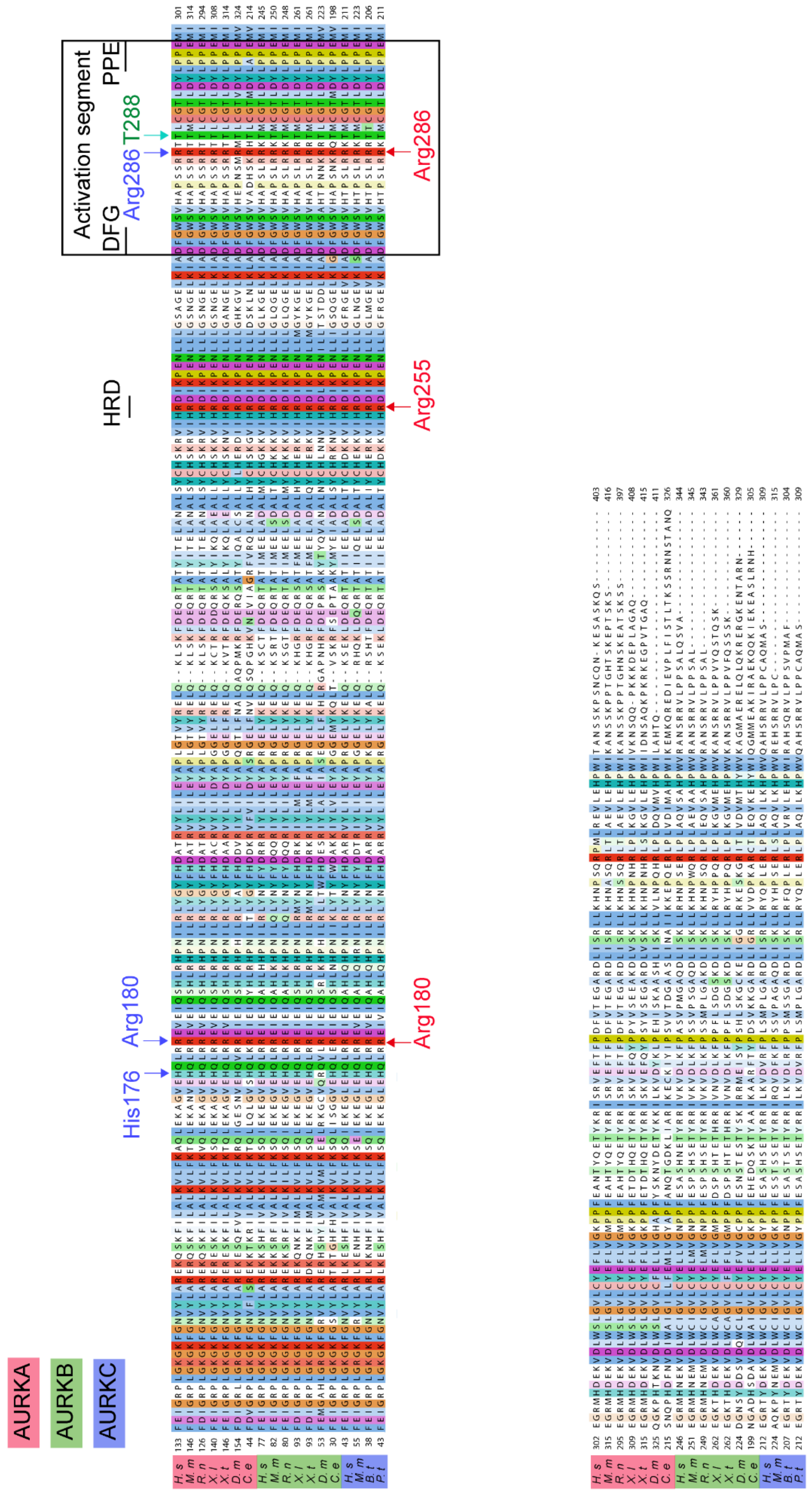

Supplementary Figure S1: Multiple protein sequence alignments of Aurora kinases A, B and C (related to Figure 1, 7 & S7)

Sequences corresponding to the kinase domains of Aurora kinases from different species include: **AURKA** from *Homo sapiens (H. s), Mus musculus (M. m), Rattus norvegicus (R. n), Xenopus laevis (X. l), Xenopus Tropicalis (X. t), Drosophila melanogaster (D.m), and Caenorhabdtitis elegans (C. e)*; **AURKB** from *Homo sapiens (H. s), Mus musculus (M. m), Rattus norvegicus (R. n), Xenopus laevis (X. l), Xenopus Tropicalis (X. t), Drosophila melanogaster (D.m),* and *Caenorhabdtitis elegans (C. e);* **AURKC** from *Homo sapiens (H. s), Mus musculus (M. m), Borus torus (B. t), Pan troglodytes (P. t).* Sequences were aligned using ClustalW and visualized with Jalview. The conserved phospho-regulatory site in the T-loop is indicated by a green arrow. Conserved residues coordinating the T-loop phospho-site are highlighted by red arrows. Conserved residues coordinating a sulfate ion in AURKA and coordinating an $INCENP^{ASS}$ phospho-site in AURKC are highlighted by blue arrows (using corresponding human AURKA residue number scheme).

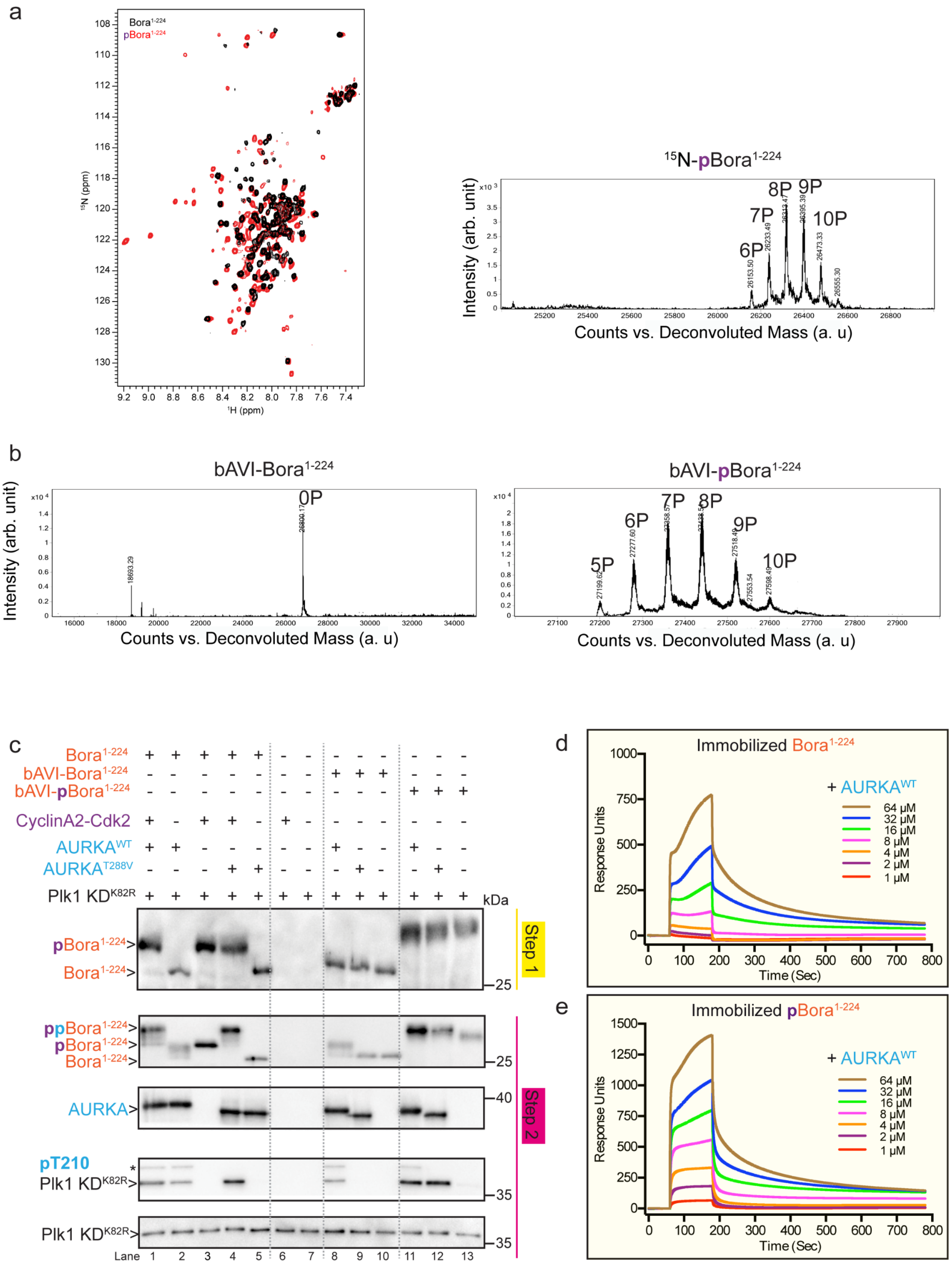
a
Bora1-224
pBora1-224
15N (ppm)
1H (ppm)
15N-pBora1-224
Intensity (arb. unit)
6P
7P
8P
9P
10P
Counts vs. Deconvoluted Mass (a. u)
b
bAVI-Bora1-224
0P
bAVI-pBora1-224
5P
6P
7P
8P
9P
10P
Intensity (arb. unit)
Counts vs. Deconvoluted Mass (a. u)
Counts vs. Deconvoluted Mass (a. u)
c
Bora1-224
bAVI-Bora1-224
bAVI-pBora1-224
CyclinA2-Cdk2
AURKAWT
AURKAT288V
Plk1 KDK82R
kDa
pBora1-224
Bora1-224
Step 1
ppBora1-224
pBora1-224
Bora1-224
AURKA
pT210
Plk1 KDK82R
Plk1 KDK82R
Step 2
Lane
d
Immobilized Bora1-224
+ AURKAWT
64 μM
32 μM
16 μM
8 μM
4 μM
2 μM
1 μM
Response Units
Time (Sec)
e
Immobilized pBora1-224
+ AURKAWT
64 μM
32 μM
16 μM
8 μM
4 μM
2 μM
1 μM
Response Units
Time (Sec)

Supplementary Figure S2: NMR analysis reveals that Bora is an intrinsically disordered protein that activates Plk1 phosphorylation by AURKA *in vitro* (related to Figure 2)

a- NMR analysis of non-phosphorylated and CyclinA2-Cdk2 phosphorylated $Bora^{1\text{-}224}$. On the left, superimposed ^{1}H-^{15}N HSQC spectra of non-phosphorylated ^{15}N-$Bora^{1\text{-}224}$ in black with CyclinA2-Cdk2 phosphorylated ^{15}N-Bora $Bora^{1\text{-}224}$ in red. On the right, intact mass spectrum of CyclinA2-Cdk2 phosphorylated ^{15}N-$Bora^{1\text{-}224}$ used in the NMR analysis at left. Arbitrary units (arb. unit), atomic mass unit (a. u.)

b- Characterization of the phosphorylation state of biotinylated AVI (bAVI)-$Bora^{1\text{-}224}$ (used in Figures 2C, 2D, 2E, 3E, 3F and supplementary Figures 2C, 2D, 2E, 3E, 3F) by intact mass spectrometry. On the left, intact mass spectrum of biotinylated AVI-$Bora^{1\text{-}224}$ (bAVI-$Bora^{1\text{-}224}$). On the right, intact mass spectrum of phosphorylated biotinylated AVI-$Bora^{1\text{-}224}$. Arbitrary units (arb. unit), atomic mass unit (a. u.).

c- Validation of the functionality of biotinylated bAVI-$pBora^{1\text{-}224}$ in a two-step Plk1 phosphorylation reaction (see **Figure 1b or 3a** for reaction schematic). Western blot analysis of kinase reactions carried out with biotinylated AVI-$Bora^{1\text{-}224}$ phosphorylated (+) or not (−) by CyclinA2-Cdk2 (step 1, highlighted in yellow) in the presence of $Plk1^{K82R}$ KD and $AURKA^{WT}$ or $AURKA^{T288V}$ (step 2, highlighted in magenta). Blots were probed with antibodies to Bora, AURKA and phosphoT210 Plk1 or pan Plk1 as indicated (from top to bottom). In the blot performed with the anti-pT210 Plk1 antibody, the asterisk denotes cross reactivity with the pT288 residue of AURKA.

d, e- SPR binding sensograms of immobilized $Bora^{1\text{-}224}$ (**d**) and $pBora^{1\text{-}224}$ (**e**) obtained with the indicated concentrations $AURKA^{WT}$ analyte. Representative profiles shown are from one experiment. See **Figures 2d and 2e** respectively for extrapolated binding plots.

Tavernier et al., Fig S3, related to Fig 3

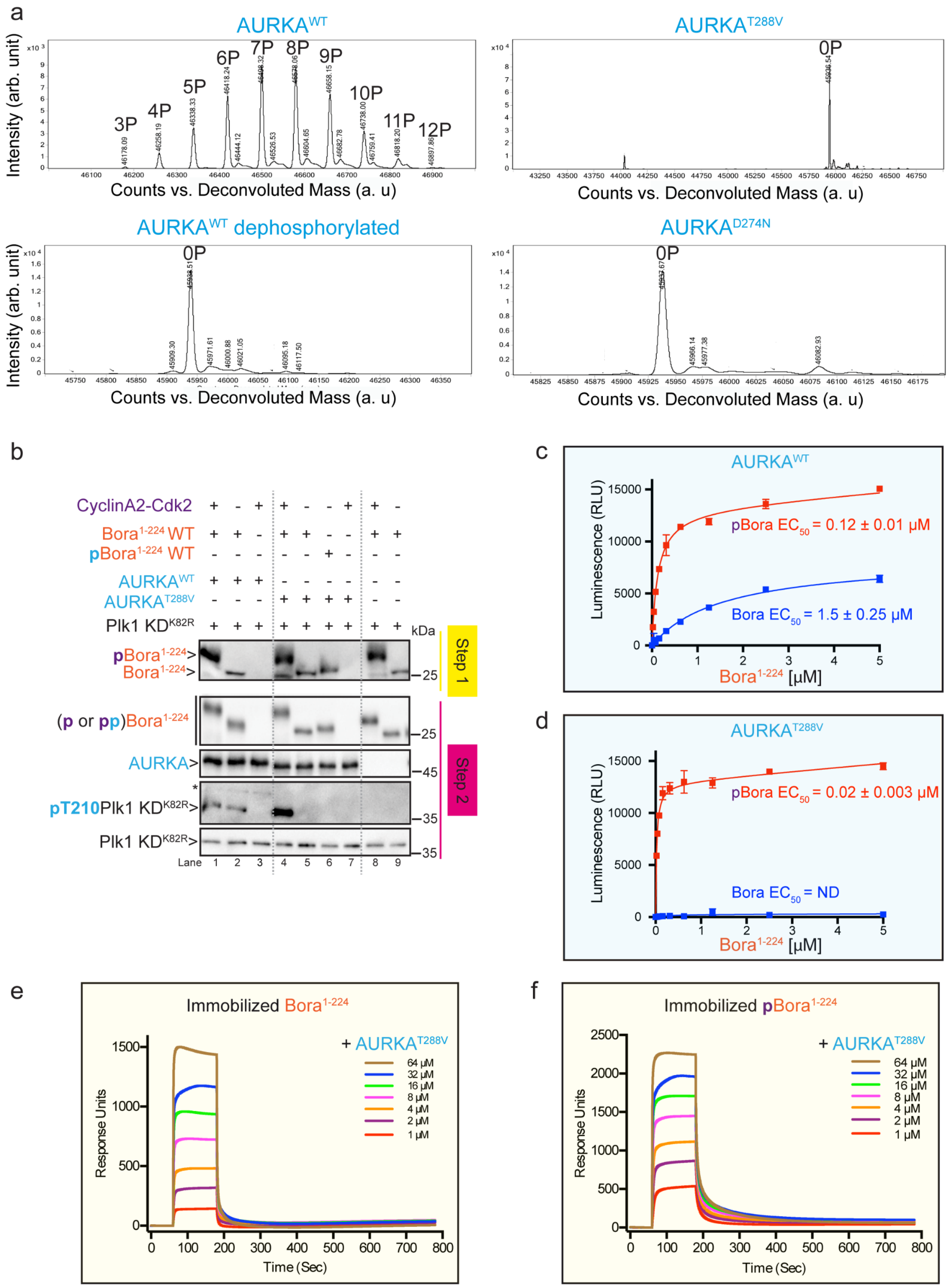

Supplementary Figure S3: CyclinA2-Cdk2-dependent phosphorylation of Bora is required for activation of AURKA^{T288V} or dephosphorylated AURKA^{WT} (related to Figure 3)

a- Intact mass spectrometry analysis of the phosphorylation state of purified recombinant AURKA^{WT} (top left), AURKA^{T288V} (top right), AURKA^{D274N} (bottom right) and AURKA^{WT} dephosphorylated with lambda phosphatase (bottom left). Arbitrary units (arb. unit), atomic mass unit (a. u.).

b- Western blot analysis of kinase reactions carried out with Plk1^{K82R} KD and AURKA^{WT} or AURKA^{T288V} (step 2, highlighted in magenta) in the presence of Bora$^{1\text{-}224}$ WT phosphorylated (+) or not (−) by CyclinA2-Cdk2 or Bora$^{1\text{-}224}$ WT pre-phosphorylated by AURKA^{WT} (pBora$^{1\text{-}224}$ WT) (step 1, highlighted in yellow). Blots were probed with antibodies to Bora, AURKA, and phosphoT210 Plk1 or pan Plk1 as indicated (from top to bottom). In the blot performed with the anti-pT210 Plk1 antibody, the asterisk denotes the cross reactivity with the pT288 residue of AURKA.

c, d- Activation of AURKA^{WT} (**c**) or AURKA^{T288V} (**d**) ATPase activity by Bora$^{1\text{-}224}$ and pBora$^{1\text{-}224}$ as assessed by the ADP Glo assay in the absence of added peptide substrate. Displayed data points and EC_{50} values represent the average luminescence (n=3, ±SD) for each reaction condition. RLU: relative light unit. ND: not determined.

e, f- SPR binding sensograms of immobilized Bora$^{1\text{-}224}$ (**e**) and pBora$^{1\text{-}224}$ (**f**) obtained with the indicated concentrations of AURKA^{T288V} analyte. Representative profiles shown are from one experiment. See **Figure 3e** and **3f** respectively for extrapolated binding plots.

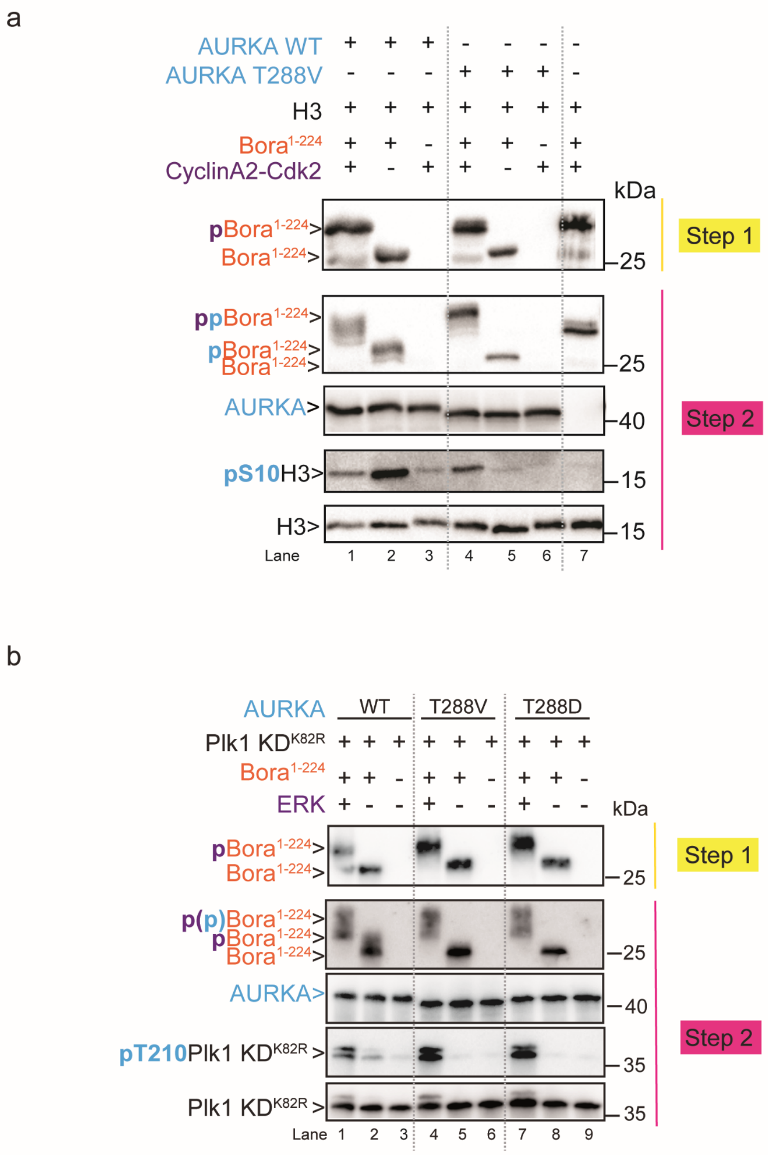
a
AURKA WT
AURKA T288V
H3
Bora1-224
CyclinA2-Cdk2
kDa
pBora1-224>
Bora1-224>
25
Step 1
ppBora1-224>
pBora1-224>
Bora1-224>
25
AURKA>
40
Step 2
pS10H3>
15
H3>
15
Lane 1 2 3 4 5 6 7
b
AURKA WT T288V T288D
Plk1 KDK82R
Bora1-224
ERK
kDa
pBora1-224>
Bora1-224>
25
Step 1
p(p)Bora1-224>
pBora1-224>
Bora1-224>
25
AURKA>
40
pT210Plk1 KDK82R >
35
Step 2
Plk1 KDK82R >
35
Lane 1 2 3 4 5 6 7 8 9

Supplementary Figure S4 (related to Figure 3)

a- Western blot analysis of 2 step kinase reactions carried out with Bora$^{1-224}$ phosphorylated (+) or not (−) by CyclinA2-Cdk2 (step 1, highlighted in yellow) in the presence of Histone H3 substrate and AURKA^{WT} or AURKA^{T288V} (step 2, highlighted in magenta). Blots were probed with antibodies to Bora, AURKA, and phosphoSer10 H3 or Histone H3 as indicated (from top to bottom).

b- Western blot analysis of 2 step kinase reactions carried out with Bora$^{1-224}$ phosphorylated (+) or not (−) by the ERK kinase (step 1, highlighted in yellow) in the presence of Plk1^{K82R} KD substrate and AURKA^{WT}, AURKA^{T288V} or AURKA^{T288D} (step 2, highlighted in magenta). Blots were probed with antibodies to Bora, AURKA, and phosphoT210 Plk1 or pan Plk1 as indicated (from top to bottom).

Tavernier at al., Fig S5, related to Fig 5

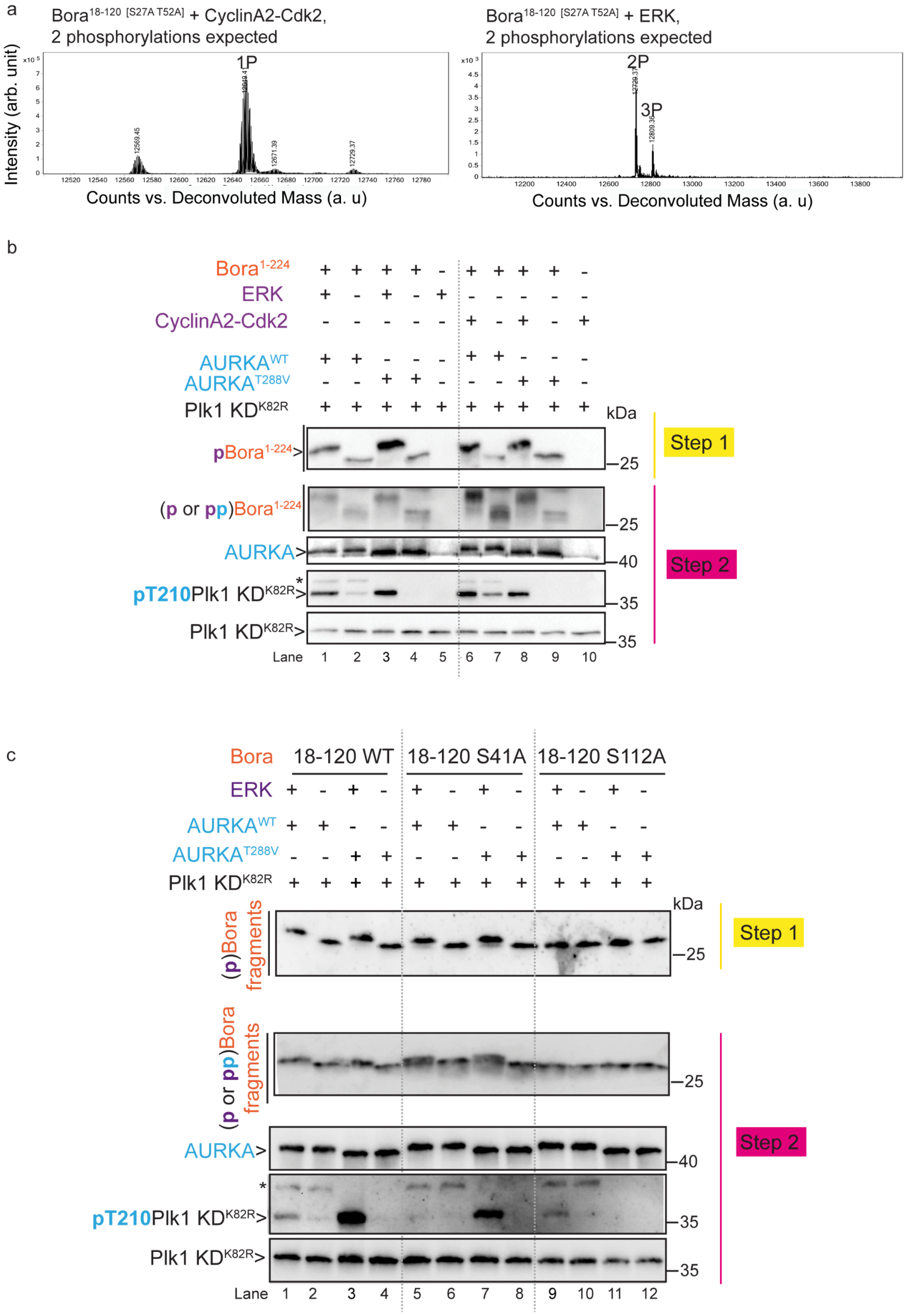

Supplementary Figure S5: ERK readily phosphorylates Bora fragments lacking Cy motifs on S/T-P sites (related to Figure 5)

a- Intact mass spectrometry analysis of the phosphorylation state of Bora$^{18\text{-}120\ [S27A\ T52A]}$ phosphorylated either by CyclinA2-Cdk2 (top panel) or by ERK (bottom panel). Arbitrary units (arb. unit), atomic mass unit (a. u.).

b- Western blot analysis of two step kinase reactions carried out with Bora$^{1\text{-}224}$ phosphorylated (+) or not (−) by CyclinA2-Cdk2 or ERK (step 1, highlighted in yellow) in the presence of Plk1^{K82R} KD and AURKA^{WT} or AURKA^{T288V} (step 2, highlighted in magenta). Blots were probed with antibodies to Bora, AURKA, and phosphoT210 Plk1 or pan Plk1 as indicated (from top to bottom). In the blot performed with the anti-pT210 Plk1 antibody, the asterisk denotes cross reactivity with the pT288 residue of AURKA.

c- Western blot analysis of two step kinase reactions carried out with Bora$^{18\text{-}120\ WT}$, Bora$^{18\text{-}120\ [S41A]}$, or Bora$^{18\text{-}120\ [S112A]}$, phosphorylated (+) or not (−) by ERK (step 1, highlighted in yellow) in the presence of Plk1^{K82R} KD and AURKA^{WT} or AURKA^{T288V} (step 2, highlighted in magenta). Blots were probed with antibodies to Bora, AURKA, and phosphoT210 Plk1 or pan Plk1 as indicated (from top to bottom).

Tavernier at al., Fig S6 related to Fig 5

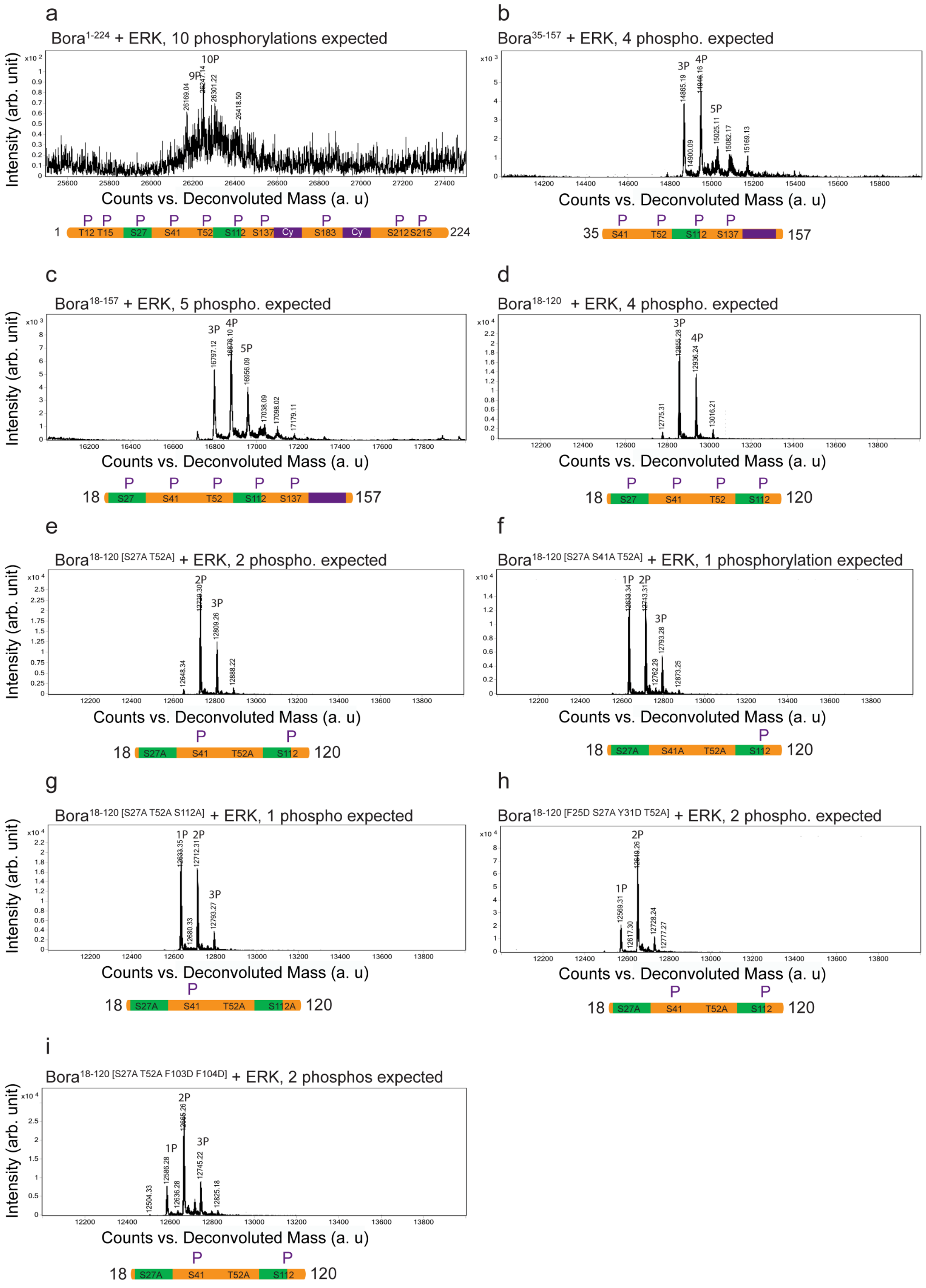

Supplementary Figure S6: Intact mass spectrometry analysis of the phosphorylation state of Bora fragments phosphorylated by ERK (related to Figure 5)

a to i- A schematic for each Bora fragment with the position of consensus S/T-P phosphorylation sites are displayed below the corresponding spectrum. Cyclin-binding motifs (Cy, in violet) and the Tpx2-like Motifs 1 or 2 (green) are indicated. Arbitrary units (arb. unit), atomic mass unit (a. u.).

Tavernier et al. Figure S7, related to Figure 7

a

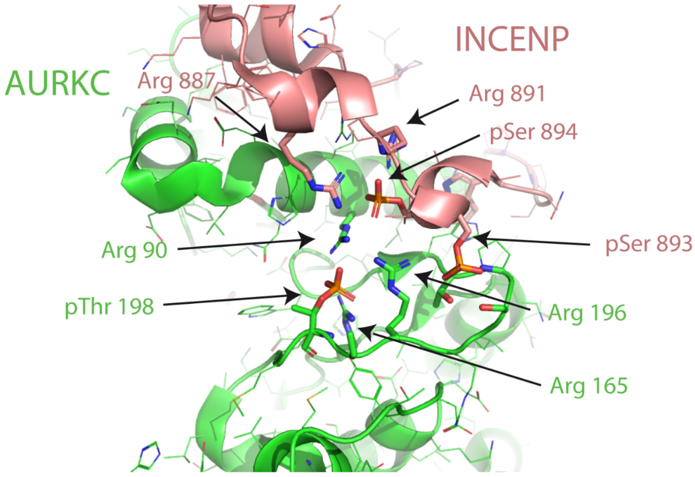

b

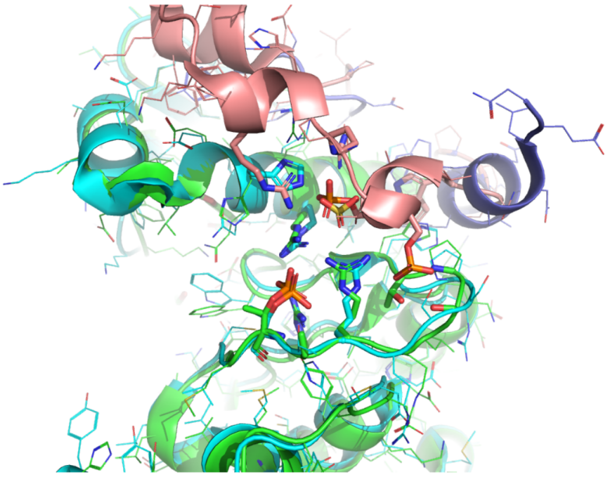

Supplementary Figure S7: Model of phospho-Bora binding to AURKA (related to Figure 7)
a- Zoom in view of the binding interface between phospho-INCENP (pink) and AURKC (green) (PDB: 6GR8 [http://DOI:10.2210/pdb6GR8/pdb]). The T-loop phospho-residue Thr198 and the phospho-T-loop coordinating residues of AURKC are highlighted in stick representation.
b- Superimposition of the crystal structures of AURKA-Tpx2 (same orientation shown in **Figure 7a**) and AURKC-phospho-INCENP (coloring is the same as in **Figure 7a** and **Supplementary Figure 7a**).

Tavernier et al. Figure S8, related to Figure 9

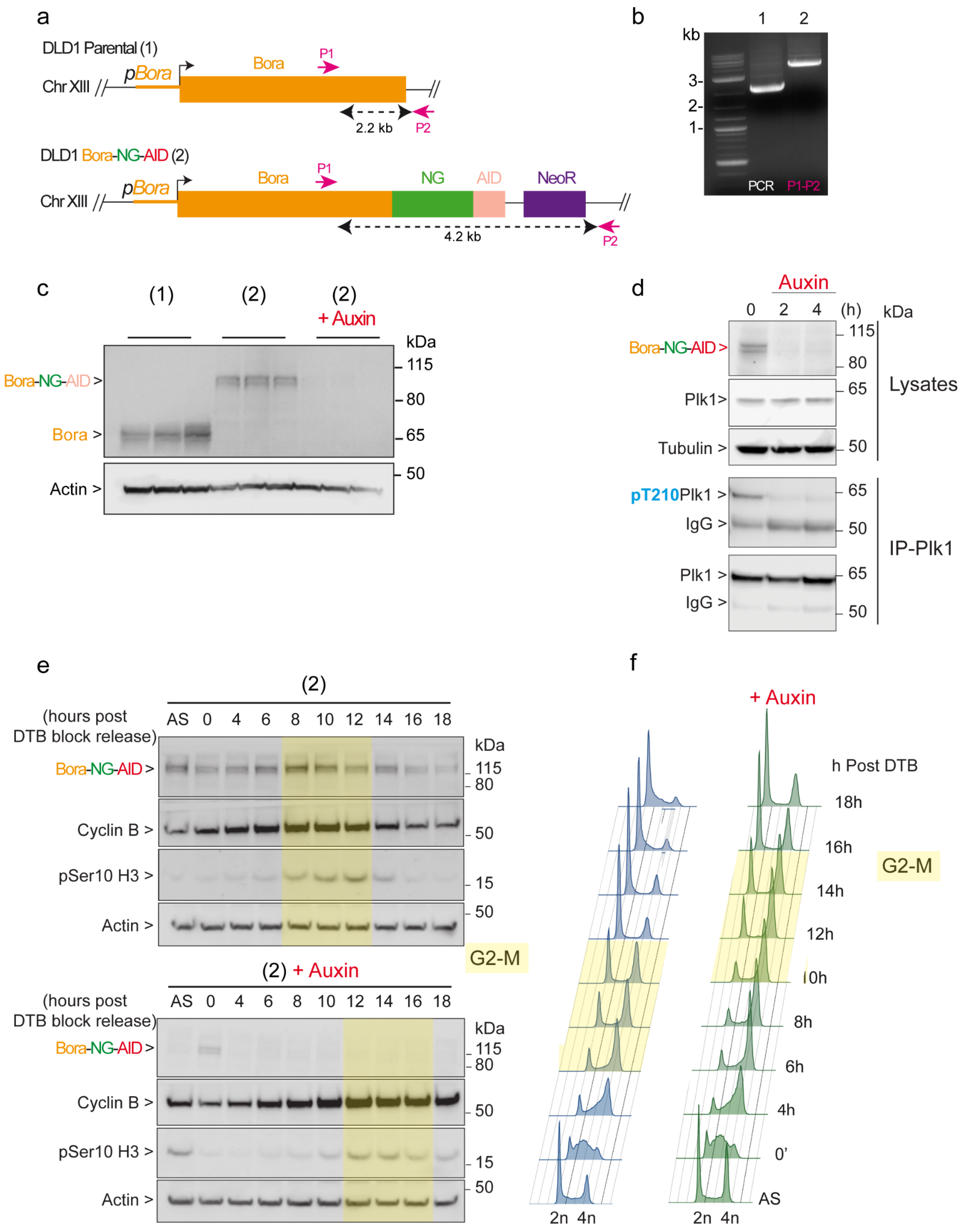

Supplementary Figure S8: Validation and characterization of the DLD1 cell line expressing endogenous Bora tagged with Neongreen (NG) and the Auxin-inducible degron (AID) (related to Figure 9)

a- Schematic of Bora gene organization in the DLD1 parental (top panel) and DLD1-Bora-Neongreen-AID cell lines (bottom panel). The position of the forward P1 and reverse P2 oligonucleotides used to genotype the cell lines is indicated.

b- PCR validation of the tag integration and determination of clone homo or heterozygosity. PCR were performed on the genomic DNA using the forward primer P1 and reverse primer P2. The successful integration leads to shift from 2 to 4.2kb visible on 1% agarose gel (lane 2). The PCR amplification was extracted and verified by DNA sequencing to confirm integration.

c- Confirmatory Western blot analyses on the selected clonal cell line DLD1 Bora-NeonGreen-AID (labelled DLD1 Bora-NG-AID for simplification) (2) and the parental DLD1 cell line (1) were performed with the indicated antibodies on 30µg protein extracts after a 24 hours treatment with Auxin.

d- DLD1 Bora-NeonGreen-AID cells were treated with Auxin for 2h and 4h, protein extracts were then submitted to immunoprecipitation (IP) with Plk1 antibodies. Lysates (input) and immunoprecipitated proteins were separated by SDS-PAGE and probed by Western blotting with the indicated antibodies.

e- DLD1 cells expressing Bora-NG-AID were synchronized by double thymidine block (G1/S phase indicated at time 0) and released intro fresh medium without (top panel) or with Auxin (bottom panel). Samples were collected at the indicated time and probed by Western blotting with the indicated antibodies. The first lane shows a lysate from asynchronous cells (AS).

f- FACS profiles of DLD1 Bora-NG-AID cells untreated or treated with Auxin after double thymidine block and release. After harvest, cells were fixed in ice cold 70% ethanol, washed and stained with PI (Propidium Iodide). FACS analyses were done using a Beckman coulter cyan ADP flow cytometer. 10 000 events were recorded in a gate within PI-Area versus PI-width dot plot, cell cycle phases were determined via flowjow using the Watson pragmatic algorithm. In panels e and f, the yellow shading on the western blots and FACS profiles highlight time points of cells in G2/M.

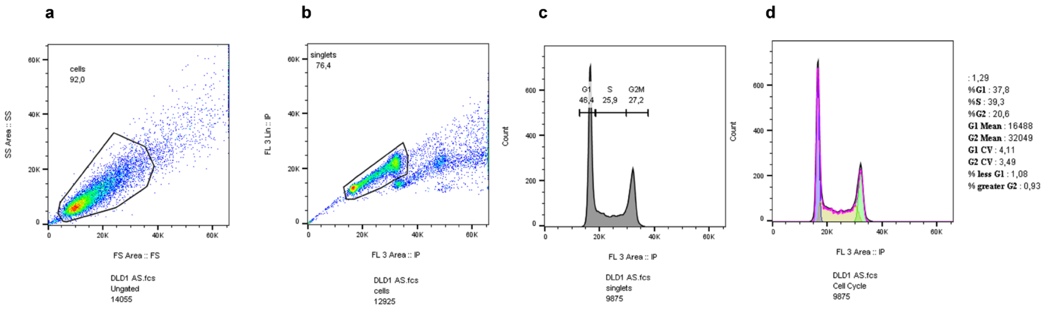

Figure S9: FACS Gating strategy
FACS analyses were done using a Beckman coulter cyan ADP flow cytometer. Gating strategy is displayed in the panel. First, we did a forward scatter (FSC) and side scatter (SSC) plot to identify single cells. Then a plot PI-Area versus PI-width to gate on singlet events and eliminate doublets. Relative percentages for gates are presented on the contour plot on the left, 10 000 events were recorded in a gate within PI-Area versus PI-width dot plot, cell cycle phases were determined via flowjo using the Watson pragmatic algorithm.

Tavernier at al., Figure S10 related to Figure 9

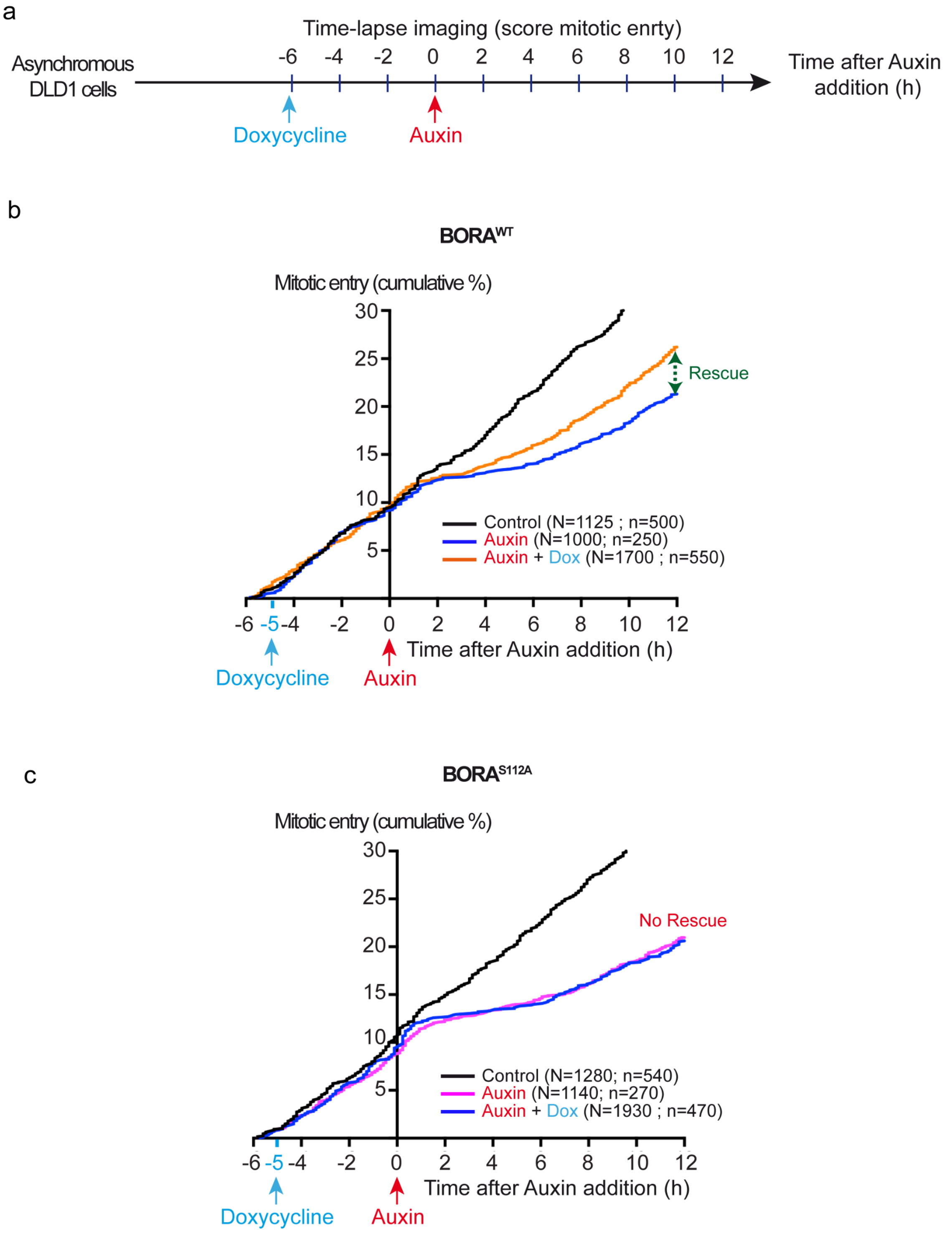

Figure S10: Entry into mitosis is rescued by exogenous Bora^{WT} but not by the Bora^{S112A} mutant (related to Figure 9)

a. Asynchronous DLD-1 Knock-In Bora-AID, RCC1-IFP2 cells stably expressing inducible Bora^{WT} or Bora^{S112A} phosphorylation mutant form, were recorded at 1 image /7 min. Entry into mitosis for each cell was defined at NEBD, using IFP2 nuclear staining. When indicated, doxycycline (blue) was added 5 hours before Auxin treatment (red). **b. c.** Graphs represent the percentage of cells that have entered into mitosis over time, normalized to cell density. N: number of cells analyzed, n: number of mitotic cells observed over time. Note that under this schedule, entry into mitosis was partially rescued by Bora^{WT} but not by Bora^{S112A}.

SUPPLEMENTARY TABLES:

Supplementary Table 1: Data collection

Ligand1	Ligand 2	Competitor	Value	Exp.	Fig
$Bora^{1-224}$	AURKA	/	Kd 52 +/- 21 μM	SPR	2D
$pBora^{1-224}$	AURKA	/	Kd 18 +/- 3 μM	SPR	2E
$Bora^{1-224}$	$AURKA^{T288V}$	/	Kd 11 +/- 2 μM	SPR	3E
$pBora^{1-224}$	$AURKA^{T288V}$	/	Kd 6 +/- 1 μM	SPR	3F
$Bora^{1-224}$ + K	AURKA	/	EC_{50} 1.2 +/- 0.7 μM	ADP-Glo	3C
$pBora^{1-224}$ + K	AURKA	/	EC_{50} 0.19 +/- 0.1 μM	ADP-Glo	3C
$pBora^{1-224}$ + K	$AURKA^{T288V}$	/	EC_{50} 0.04 +/- 0.001 μM	ADP-Glo	3D
$Bora^{1-224}$	AURKA	/	EC_{50} 1.5 +/- 0.25 μM	ADP-Glo	S3C
$pBora^{1-224}$	AURKA	/	EC_{50} 0.12+/- 0.01 μM	ADP-Glo	S3C
$pBora^{1-224}$	$AURKA^{T288V}$	/	EC_{50} 0.02 +/- 0.003 μM	ADP-Glo	S3D
FITC-$Tpx2^{1-43}$	AURKA	/	Kd 0.009 +/- 0.0005 μM	FP	6B
FITC-$Tpx2^{1-43}$	AURKA	$Tpx2^{1-43}$	IC_{50} 0.1 +/- 0.015 μM	FP	6C
FITC-$Tpx2^{1-43}$	AURKA	$pBora^{1-224}$	IC_{50} 2.7 +/- 1.3 μM	FP	6C
FITC-$Tpx2^{1-43}$	$AURKA^{T288V}$	/	Kd 0.015 +/- 0.0006 μM	FP	6D
FITC-$Tpx2^{1-43}$	$AURKA^{T288V}$	$Tpx2^{1-43}$	IC_{50} 0.11 +/- 0.005 μM	FP	6E
FITC-$Tpx2^{1-43}$	$AURKA^{T288V}$	$pBora^{1-224}$	IC_{50} 0.032 +/- 0.004 μM	FP	6E
FITC-$Tpx2^{1-43}$	$AURKA^{T288V}$	$Bora^{1-224}$	IC_{50} 0.73 +/- 0.13 μM	FP	6E
FITC-$Tpx2^{1-43}$	$AURKA^{T288V}$	$pBora^{1-224}$	IC_{50} 0.036 +/- 0.002 μM	FP	6F
FITC-$Tpx2^{1-43}$	$AURKA^{T288V}$	$pBora^{18-120\ [S27A\ T52A]}$	IC_{50} 0.68 +/- 0.09 μM	FP	6F
$pBora^{1-224}$ + K	AURKA	/	EC_{50} 3.8 +/- 1,8 μM	ADP-Glo	6G
$Bora^{1-224}$ + K	AURKA	/	EC_{50} 0.19 +/- 0.06 μM	ADP-Glo	6G
$Tpx2^{1-43}$ + K	AURKA	/	EC_{50} 0.01 +/- 0.001 μM	ADP-Glo	6G
$pBora^{1-224}$ + K	$AURKA^{T288V}$	/	EC_{50} 0.026 +/- 0.0015 μM	ADP-Glo	6H
$pBora^{1-224}$ + K	$AURKA^{T288V}$	$Tpx2^{1-43}$	IC_{50} 0.46 +/- 0.04 μM	ADP-Glo	6I
FITC-$Tpx2^{1-43}$	$AURKA^{T288V}$	/	Kd 9.9 +/- 0.88 nM	FP	7B
FITC-$Tpx2^{1-43}$	$AURKA^{T288V\ R255A}$	/	Kd 36 +/- 2 nM	FP	7B
FITC-$Tpx2^{1-43}$	$AURKA^{T288V\ R180A}$	/	Kd 5.6 +/- 0.6 nM	FP	7B
FITC-$Tpx2^{1-43}$	$AURKA^{T288V\ R286A}$	/	Kd 8.5 +/- 1.2 nM	FP	7B
FITC-$Tpx2^{1-43}$	$AURKA^{T288V\ H176A}$	/	Kd 8.2 +/- 0.68 nM	FP	7B
FITC-$Tpx2^{1-43}$	$AURKA^{T288V}$	$pBora^{1-224}$	IC_{50} 35 +/- 1.3 nM	FP	7D
FITC-$Tpx2^{1-43}$	$AURKA^{T288V\ R255A}$	$pBora^{1-224}$	IC_{50} 771 +/- 179 nM	FP	7D
FITC-$Tpx2^{1-43}$	$AURKA^{T288V\ R180A}$	$pBora^{1-224}$	IC_{50} 720 +/- 180 nM	FP	7D
FITC-$Tpx2^{1-43}$	$AURKA^{T288V\ R286A}$	$pBora^{1-224}$	IC_{50} 516 +/- 77 nM	FP	7D
FITC-$Tpx2^{1-43}$	$AURKA^{T288V\ H176A}$	$pBora^{1-224}$	IC_{50} 290 +/- 26 nM	FP	7D

K: Kemptide peptide
FP: Fluorescence Polarization

Supplementary Table 2: Plasmids used in this study

Backbone	Insert	Cloning methods	ID/Source
pDONR201	6xHis-TEV-Bora$^{1-224}$	Gateway	pLP1848
pDEST17	6xHis-TEV-Bora$^{1-224}$	Gateway	pLP1850
pDONR201	6xHis-TEV-AVI-Bora$^{1-224}$	Gateway	pLP2006
pDEST17	6xHis-TEV-AVI-Bora$^{1-224}$	Gateway	pLP2009
pDONR201	6xHis-TEV-Bora$^{18-157}$	Gateway	pLP2049
pDEST17	6xHis-TEV-Bora$^{18-157}$	Gateway	pLP2053
pDONR201	6xHis-TEV-Bora$^{18-120}$	Gateway	pLP2072
pDEST17	6xHis-TEV-Bora$^{18-120}$	Gateway	pLP2076
pDEST17	6xHis-TEV-Bora$^{18-120\ [S27A\ T52A]}$	SDM* pLP2076	pLP2078
pDONR201	6xHis-TEV-Bora$^{35-157}$	Gateway	pLP2073
pDEST17	6xHis-TEV-Bora$^{35-157}$	Gateway	pLP2077
pDEST17	6xHis-TEV-Bora$^{18-120\ [S27A\ S41A\ T52A]}$	SDM pLP2076	pNT368
pDEST17	6xHis-TEV Bora$^{18-120\ [S27A\ T52A\ S112A]}$	SDM pLP2076	pNT369
pDEST17	6xHis-TEV Bora$^{18-120\ [S41A]}$	SDM pLP2076	pLP2456
pDEST17	6xHis-TEV Bora$^{18-120\ [S112A]}$	SDM pLP2076	pLP2446
pDEST17	6xHis-TEV Bora$^{18-120\ [F25D\ S27A\ Y31D\ T52A]}$	SDM pLP2076	pLP2225
pDEST17	6xHis-TEV Bora$^{18-120\ [S27A\ T52A\ F103D\ F104D]}$	SDM pLP2076	pLP2214
pETM30-2	6xHis-GST-TEV-AURKA	CRE°	pNT256
pPROEXHTa	6xHis-TEV-Cyclin A2		pNT217
pGEX4T-3	GST-Cdk2 GST-Cak1		T. Mittag
pET	ERK2-MEK_RF4		Addgene#39212
pET-28b(+)	AURKA-6xHis		E. Nigg
pETM30-2	6xHis-GST-TEV-AURKA^{T288V}	SDM pNT256	pNT257
pETM30-2	6xHis-GST-TEV-AURKA^{T288D}	SDM pNT256	pLP2445
pETM30-2	6xHis-GST-TEV-AURKA^{D274N}	SDM pNT256	pNT351
pETM30-2	6xHis-GST-TEV-AURKA$^{[T288V\ R255A]}$	SDM pNT257	pLP2371
pETM30-2	6xHis-GST-TEV-AURKA$^{[T288V\ R180A]}$	SDM pNT257	pLP2372
pETM30-2	6xHis-GST-TEV-AURKA$^{[T288V\ R286A]}$	SDM pNT257	pLP2373
pETM30-2	6xHis-GST-TEV-AURKA$^{[T288V\ H176A]}$	SDM pNT257	pLP2374
pGEX-2T	GST-TEV-Plk1	CRE	pNT87
pGEX-2T	GST-TEV-Plk1^{K82R}	SDM pNT87	pNT179
pGEX-2T	GST-TEV-Plk1$^{1-370\ [K82R]}$	CRE pNT179	pNT287
pGEX-2T	GST-TEV-Plk1$^{1-330\ [K82R]}$	CRE pNT179	pNT284
pGEX-2T	GST-TEV-Plk1$^{1-330}$	CRE pNT179	pNT274
pX330	3xFlag-hSpCas9		Addgene#42230
pX330	3xFlag-hSpCas9+gRNA AAVS1		Addgene#72833
pX330	3xFlag-hSpCas9+gRNA Bora Exon 12		pLP1852
pMK243	Empty		Addgene#72835
pMK243	Bora	CRE	pLP2126
pMK243	Bora$^{[S41A\ S122A\ S137A]}$	CRE	pLP2158
pMK243	Bora^{S41A}	CRE	pLP2165
pMK243	Bora^{S112A}	CRE	pLP2136
pMK243	Bora^{S137A}	CRE	pLP2166
pMK243	Bora$^{\Delta35}$	CRE	pLP2157
pMK243	Bora$^{[F25D\ Y31D]}$	CRE	pLP2227
pMK243	Bora$^{[F103D\ F104D]}$	CRE	pLP2247

*SDM: Site-directed Mutagenesis, °CRE: Cloning by restriction enzymes

Supplementary Table 3: **List of primers used in this study**

Purpose and sequences	ID
Forward and reverse primers to clone TEV-Bora$^{1-224}$ Gateway:<br>GGGGACAAGTTTGTACAAAAAAGCAGGCTTCCCAACGACCGAAAACCTGTATTTTCAGGGCGC CATGGATCCGGAATTCATGGGAGATGTCAAGGAATC-F<br>GGGGACCACTTTGTACAAGAAAGCTGGGTCTTATAGTGATGTTTGAACACC-R	oLP1655<br>oLP1656
Forward and reverse primers to clone TEV-AVI-Bora$^{1-224}$ Gateway:<br>GGGGACAAGTTTGTACAAAAAAGCAGGCTTCCCAACGACCGAAAACCTGTATTTTCAGGGCGG CTTAAACGATATTTTTGAAGCGCAGAAAATTGAATGGCATGAAATGGGAGATGTCAAGGAATC-F<br>GGGGACCACTTTGTACAAGAAAGCTGGGTCTTATAGTGATGTTTGAACACC-R	oLP1923<br>oLP1656
Forward and reverse primers for Bora^{S27A}:<br>GTTTTAAATCCTTTTGAAGCTCCTAGTGATTATTCTAATCTCCATG-F<br>CATGGAGATTAGAATAATCACTAGGAGCTTCAAAAGGATTTAAAAC-R	oNT168<br>oNT169
Forward and reverse primers for Bora^{S41A}:<br>CTCCATGAACAAACTCTCGCCgctCCTTCTGTTTTTAAATCAAC-F<br>GTTGATTTAAAAACAGAAGGagcGGCGAGAGTTTGTTCATGGAG-R	oNT125<br>oNT126
Forward and reverse primers for Bora^{T52A}:<br>GTTTTTAAATCAACAAAATTACCAGCTCCAGGGAAATTTAGATGG-F<br>CCATCTAAATTTCCCTGGAGCTGGTAATTTTGTTGATTTAAAAAC-R	oNT170<br>oNT171
Forward and reverse primers for Bora^{S112A}:<br>GTTTTTCACTAAAGATGTCATCGTACCCgctCCTTGGACTGATCATG-F<br>CATGATCAGTCCAAGGAGCGGGTACGATGACATCTTTAGTGAAAAAC-R	oNT127<br>oNT128
Forward and reverse primers for Bora^{S137A}:<br>GCACTAACATAAATAGTGACGCTCCAGTTGGAAAAAAGCTGACC-F<br>GGTCAGCTTTTTTCCAACTGGAGCGTCACTATTTATGTTAGTGC-R	oNT129<br>oNT130
Forward and reverse primers to clone TEV-Bora$^{18-120}$ by Gateway:<br>GGGGACAAGTTTGTACAAAAAAGCAGGCTTCCCAACGACCGAAAACCTGTATTTTCAGGGCGC CATGGATCCGGAATTCAGGATCCCTGTTTTAAATCCTTTTG-F<br>GGGGACCACTTTGTACAAGAAAGCTGGGTCTTATTTCCCTTCATGATCAGTCCAAG-R	oNT326<br>oNT329
Forward and reverse primers to clone TEV-Bora$^{35-157}$ by Gateway:<br>GGGGACAAGTTTGTACAAAAAAGCAGGCTTCCCAACGACCGAAAACCTGTATTTTCAGGGCGC CATGGATCCGGAATTCCATGAACAAACTCTCGCCAG-F<br>GGGGACCACTTTGTACAAGAAAGCTGGGTCTTACAATGTCTGACAAGCAGCATCG-R	oNT328<br>oNT327
Forward and reverse primers for Bora$^{[F25D\ Y31D]}$:<br>CCTGTTTTAAATCCTGATGAAAGTCCTAGTGATGATTCTAATCTCCATGAACAAACTCTCG-F<br>CGAGAGTTTGTTCATGGAGATTAGAATCATCACTAGGACTTTCATCAGGATTTAAAACAGG-R	oLP2126<br>oLP2127
Forward and reverse primers for Bora$^{[F103D\ F104D]}$:<br>GACAAAAGACAAAAAGCCATTGAAGAGGATGACACTAAAGATGTCATCGTACCCTCTCC-F<br>GGAGAGGGTACGATGACATCTTTAGTGTCATCCTCTTCAATGGCTTTTTGTCTTTTGTC-R	oLP2157<br>oLP2128
Forward and reverse primers to amplify and clone AURKA in pETM30 NcoI/XhoI :<br>CGCCATGGACCGATCTAAAGAAAAC-F<br>GGCCTCGAGCTAAGACTGTTTGCTAGCTG-R	oNT37<br>oNT38
Forward and reverse primers for AURKA^{T288V}:<br>GCTCCATCCTCCAGGAGGACCGTCCTCTGTGGCACCCTGGACTACC-F<br>GGTAGTCCAGGGTGCCACAGAGGACGGTCCTCCTGGAGGATGGAGC-R	oNT123<br>oNT124
Forward and reverse primers for AURKA^{T288D}:<br>CATGCTCCATCCTCCAGGAGGACCGACCTCTGTGGCACCCTGGACTACC-F<br>GGTAGTCCAGGGTGCCACAGAGGTCGGTCCTCCTGGAGGATGGAGCATG-R	oLP2582<br>oLP2583
Forward and reverse primers for AURKA^{D274N}:<br>GAGCTTAAAATTGCAAATTTTGGGTGGTCAGTACATG-F<br>CATGTACTGACCACCCAAAATTTGCAATTTTAAGCTC-R	oNT324<br>oNT325
Forward and reverse primers for AURKA^{H176A}:<br>GGAGAAAGCCGGAGTGGAGGCTCAGCTCAGAAGAGAAGTAG-F<br>CTACTTCTCTTCTGAGCTGAGCCTCCACTCCGGCTTTCTCC-R	oLP2467<br>oLP2468

Forward and reverse primers for AURKA^{R255A}:<br>CGAAGAGAGTTATTCATGCAGACATTAAGCCAGAG-F<br>CTCTGGCTTAATGTCTGCATGAATAACTCTCTTCG-R	oLP2461<br>oLP2462
Forward and reverse primers for AURKA^{R180A}:<br>GGAGTGGAGCATCAGCTCAGAGCAGAAGTAGAAATACAGTCCC-F<br>GGGACTGTATTTCTACTTCTGCTCTGAGCTGATGCTCCACTCC-R	oLP2463<br>oLP2464
Forward and reverse primers for AURKA $^{T288V\ R286A}$:<br>CATGCTCCATCCTCCAGGGCGACCGTCCTCTGTGGCACCC-F<br>GGGTGCCACAGAGGACGGTCGCCCTGGAGGATGGAGCATG-R	oLp2465<br>oLP2466
Forward and reverse primers to clone Plk1 in pGEX-2T EcoRI/XhoI:<br>CGCGAATTCATGAGTGCTGCAGTGACTGCAG-F<br>CGCCTCGAGTTAGGAGGCCTTGAGACGGTTGC-R	oLP2467<br>oLP2468
Forward and reverse primers to clone Plk1$^{1\text{-}370}$ in pGEX-2T EcoRI/XhoI:<br>CGCGAATTCATGAGTGCTGCAGTGACTGCAG-F<br>CGCCTCGAGTTAGACCACCTCACCTGTCTCTC-R	oNT2<br>oNT3
Forward and reverse primers clone Plk1$^{1\text{-}330}$ in pGEX-2T EcoRI/XhoI:<br>CGCGAATTCATGAGTGCTGCAGTGACTGCAG-F<br>CGCCTCGAGTTAGCTGGGAGCAATCGAAAACCTTG-R	oNT2<br>oNT189
Forward and reverse primers for Plk1^{K82R}:<br>GAGGTGTTCGCGGGCCGGATTGTGCCTAAGTCTCTGCT-F<br>AGCAGAGACTTAGGCACAATCCGGCCCGCGAACACCTC-R	oNT118<br>oNT119
GuideRNA#1 and #2taregting exon 12 in Bora:<br>Guide#1: 5' CACACAACACAGAGGTGTTGG-F<br>Guide#2: 5' GGACTGCTGCATTGAAAAGGG-R	oLP1625<br>oLP1758
Forward and reverse primers to amplify GuideRNA#1 targeting Bora exon 12 to be subcloned into PX330-U6-hSpCas9<br>CACCGCACACAACACAGAGGTGT-F<br>AAACACACCTCTGTGTTGTGTGC-R	Guide 1-1<br>Guide 1-2
Forward and reverse primers to amplify GuideRNA#2 targeting Bora exon 12 to be subcloned into PX330-U6-hSpCas9<br>CACCGGACTGCTGCATTGAAAA-F<br>AAACTTTTCAATGCAGCAGTCC-R	Guide 2-1<br>Guide 2-2
Forward and reverse primers to screen the integration of the AID cassette in Bora exon 12<br>TACGCAGAATTGTGGAAGCA (P1)-F<br>AGCCCCACTTTTTCTCTTGG (P2)-R	P1/P2
Forward and reverse primers to clone Bora full length in pMK243 SalI/BglII<br>CGGCCGCACGCGTGTCGACATGGGAGATGTCAAGGAAT-F<br>CTGCAGAGATCTTCACTATGGACTGCTGCATTG-R	oLP2066<br>oLP2067
Forward and reverse primers to clone Bora starting at 35 in pMK243 MluI/BglII<br>CGGCCGCACGCGTGTCGACATGCATGAACAAACTCTCGCCAGTCC-F<br>CTGCAGAGATCTTCACTATGGACTGCTGCATTG-R	oLP2107<br>oLP2067
