# Supplementary material for: Bora phosphorylation substitutes in trans for T-loop phosphorylation in Aurora A to promote mitotic entry
Source: Nat Commun. 2021 Mar 26;12:1899. doi: 10.1038/s41467-021-21922-w (PMC7997955; doi:10.1038/s41467-021-21922-w)
Supplement: Supplementary file 3 — Reporting Summary [file 41467_2021_21922_MOESM3_ESM.pdf]

nature research

Corresponding author(s): Frank Sicheri & Lionel Pintard

Last updated by author(s): Feb 11, 2021

Reporting Summary

Nature Research wishes to improve the reproducibility of the work that we publish. This form provides structure for consistency and transparency in reporting. For further information on Nature Research policies, see our Editorial Policies and the Editorial Policy Checklist.

Statistics

For all statistical analyses, confirm that the following items are present in the figure legend, table legend, main text, or Methods section.

n/a	Confirmed	
☐	☒	The exact sample size (*n*) for each experimental group/condition, given as a discrete number and unit of measurement
☐	☒	A statement on whether measurements were taken from distinct samples or whether the same sample was measured repeatedly
☒	☐	The statistical test(s) used AND whether they are one- or two-sided<br>*Only common tests should be described solely by name; describe more complex techniques in the Methods section.*
☒	☐	A description of all covariates tested
☒	☐	A description of any assumptions or corrections, such as tests of normality and adjustment for multiple comparisons
☐	☒	A full description of the statistical parameters including central tendency (e.g. means) or other basic estimates (e.g. regression coefficient) AND variation (e.g. standard deviation) or associated estimates of uncertainty (e.g. confidence intervals)
☒	☐	For null hypothesis testing, the test statistic (e.g. *F*, *t*, *r*) with confidence intervals, effect sizes, degrees of freedom and *P* value noted<br>*Give P values as exact values whenever suitable.*
☒	☐	For Bayesian analysis, information on the choice of priors and Markov chain Monte Carlo settings
☒	☐	For hierarchical and complex designs, identification of the appropriate level for tests and full reporting of outcomes
☒	☐	Estimates of effect sizes (e.g. Cohen's *d*, Pearson's *r*), indicating how they were calculated

Our web collection on statistics for biologists contains articles on many of the points above.

Software and code

Policy information about availability of computer code

Data collection	NMR: 600-MHz Bruker AVANCE III spectrometers equipped with a 1.7-mm TCI CryoProbe<br>SPR: Biacore S200<br>ADPglo: Biotek Synergy Neo plate reader (BioTek)<br>Fluo polarization: BioTek Synergy Neo plate reader (BioTek)<br>FACS: Beckman coulter cyan ADP flow cytometer<br>Mass spectrometry: 1260 series HPLC connected to 6538 UHD series electrospray ionization quadrupole time-of-flight mass spectrometer Agilent<br>Microscopy: inverted microscope (Nikon Eclipse TI-E) controlled by Metamorph software and equipped with perfect focus system, fast emission filter wheel (lambda 10-3, Sutter), electron multiplying charge coupled device camera (iXon 3 888 Ultra Andor), Plan Apochromat 20x /NA 0.75 lens, and light-emitting diode (LED)-based illumination system (spectra X-light engine, Lumencor). Filters used for IFP2 were ET620/60x, T660lpxr beamsplitter and ET700/75m from Chroma.
Data analysis	NMR: data processing was conducted using NMRviewJ and NMRpipe and NMR spectra were analyzed using Analysis<br>SPR: Biacore S200 analysis software (Cytiva)<br>ADPGlo: GraphPad Prism V8.4.2<br>Fluo polarization: Gen5 v2.05 software and GraphPad Prism V8.4.2<br>FACS: Summit 4.3 acquisition software but were then processed and analyzed with Flowjo (BD) using the Watson, pragmatic algorithm.<br>Mass spectrometry: Agilent MassHunter software version B.06.01<br>Microscopy: ImageJ

For manuscripts utilizing custom algorithms or software that are central to the research but not yet described in published literature, software must be made available to editors and reviewers. We strongly encourage code deposition in a community repository (e.g. GitHub). See the Nature Research guidelines for submitting code & software for further information.

Data

Policy information about availability of data

All manuscripts must include a data availability statement. This statement should provide the following information, where applicable:

- Accession codes, unique identifiers, or web links for publicly available datasets
- A list of figures that have associated raw data
- A description of any restrictions on data availability

Authors confirm that all relevant data are included in the paper and/or its supplementary information files

Field-specific reporting

Please select the one below that is the best fit for your research. If you are not sure, read the appropriate sections before making your selection.

☒ Life sciences ☐ Behavioural & social sciences ☐ Ecological, evolutionary & environmental sciences

For a reference copy of the document with all sections, see nature.com/documents/nr-reporting-summary-flat.pdf

Life sciences study design

All studies must disclose on these points even when the disclosure is negative.

Sample size	Statistical methods were not used to determine sample sizes.<br><br>However, for quantification purposes, a sample size of n = 3 unless otherwise stated was used to assess reproducibility and robustness of each experiment performed. Sample sizes were based on prior experience in the field.
Data exclusions	No data was excluded from analysis
Replication	To ensure reproducibility of experimental findings, all biochemical or cellular assays were repeated independently at least three times. One representative result for each experiment is presented in the main figures or the supplementary figures. The results from all experiments repeating those in the paper were essentially the same as those presented in the paper.
Randomization	Randomization for experiments was not relevant because all protein samples and cells used for analysis were from the same initial stocks.
Blinding	Blinding for experiments was not relevant because all data collection and analysis were quantitative and not qualitative in nature

Reporting for specific materials, systems and methods

We require information from authors about some types of materials, experimental systems and methods used in many studies. Here, indicate whether each material, system or method listed is relevant to your study. If you are not sure if a list item applies to your research, read the appropriate section before selecting a response.

Materials & experimental systems

n/a	Involved in the study	
☐	☒	Antibodies
☐	☒	Eukaryotic cell lines
☒	☐	Palaeontology and archaeology
☐	☒	Animals and other organisms
☒	☐	Human research participants
☒	☐	Clinical data
☒	☐	Dual use research of concern

Methods

n/a	Involved in the study	
☒	☐	ChIP-seq
☐	☒	Flow cytometry
☒	☐	MRI-based neuroimaging

Antibodies

Antibodies used

anti-human Plk1 (Abcam Cat#ab17057),
anti-human Plk1 (Bethyl Cat#A300-250A),
anti-Bora (Cell Signaling Technologies Cat#D2B9),
anti-Bora (Santa-Cruz Cat#sc-393741),
anti-Phospho-Histone H3 (Ser10) (Cell Signaling, Technologies Cat#9701),
anti-Phospho-Plk1 (Thr210) (Cell Signaling, Technologies Cat#5412),
anti-Actin (MP Biomedical Cat#69100),
anti-Tubulin – DM1A (MerckMillipore Cat#T9026),

anti-AURKA/Aurora A (Cell Signaling Technologies Cat#91590),
anti-human Greatwall (Burgess et al., 2010),
anti-Xenopus Cdc25 (Lorca et al., 2010),
anti-Xenopus Plx1 (Vigneron et al., 2018),
anti-Phospho-PP1 (Thr320) (Abcam Cat#abab62334),
anti-Phospho-Cdc2 (Tyr15) (Cell Signaling Technologies Cat#9111),
anti-ERK (Abrieu et al., 1996),
anti-Phospho-ERK (Cell Signaling Technologies Cat#9106S),
anti-Xenopus Bora (Vigneron et al., 2018),
anti-Cyclin B1 (Cell Signaling Technologies Cat#D5C10),
Peroxidase Goat Anti-Mouse IgG (H+L) (MerckMillipore Cat#A9917),
Peroxidase Goat Anti-Rabbit IgG (H+L) (MerckMillipore Cat#A0545),
TrueBlot ULTRA (Rockland Cat#18-8817-30).
Antibodies all of themwere diluted at 1/1000e.

Validation: Antibodies were validated for their specificity by the source companies / suppliers

Eukaryotic cell lines

Policy information about cell lines

Cell line source(s)	ATTCC DLD1 CCL-221
Authentication	Not authenticated
Mycoplasma contamination	All tested negative for mycoplasma
Commonly misidentified lines (See ICLAC register)	No commonly misidentified cell lines were used in the study.

Animals and other organisms

Policy information about studies involving animals; ARRIVE guidelines recommended for reporting animal research

Laboratory animals	Xenopus leavis, females, 6 to 10 years of age
Wild animals	Study did not involve wild animals
Field-collected samples	The study did not involve samples collected from the field
Ethics oversight	French Government. Approval n° B34-172-39 and Ministère de L'Enseignement Supérieur de la l'Innovation of France (Approval n° APAFIS#4971-2016041415177715v4).

Note that full information on the approval of the study protocol must also be provided in the manuscript.

Flow Cytometry

Plots

Confirm that:

- [x] The axis labels state the marker and fluorochrome used (e.g. CD4-FITC).
- [x] The axis scales are clearly visible. Include numbers along axes only for bottom left plot of group (a 'group' is an analysis of identical markers).
- [x] All plots are contour plots with outliers or pseudocolor plots.
- [x] A numerical value for number of cells or percentage (with statistics) is provided.

Methodology

Sample preparation	Cells were fixed in ice cold 70% ethanol, washed and stained with 50 µg/ml PI (Propidium Iodide) and 50 µg/ml RNase A
Instrument	FACS analyses were done using a Beckman coulter cyan ADP flow cytometer
Software	The data were acquired using Summit 4.3 acquisition software but were then processed and analyzed with Flowjo (BD) using the Watson, pragmatic algorithm.
Cell population abundance	Among all the samples analyzed from 80 to 90% of the cell population was composed of live cells (no debris, no dead cells), within this cell population we identified around 80% of singlets.
Gating strategy	We measured the forward scatter (FSC) and side scatter (SSC) to identify single cells and exclude debris and dead cells.

Gating strategy

10 000 events were then recorded in a gate within PI-Area versus PI-width dot plot (to exclude clumps and doublets)

☒ Tick this box to confirm that a figure exemplifying the gating strategy is provided in the Supplementary Information.
